# Ciclopirox Hydroxypropyl Chitosan (CPX-HPCH) Nail Lacquer and Breathable Cosmetic Nail Polish: In Vitro Evaluation of Drug Transungual Permeation Following the Combined Application

**DOI:** 10.3390/life12060801

**Published:** 2022-05-27

**Authors:** Daniela Monti, Silvia Tampucci, Valentina Paganini, Susi Burgalassi, Patrizia Chetoni, Jordi Galván, Francesco Celandroni, Emilia Ghelardi

**Affiliations:** 1 Department of Pharmacy, University of Pisa, 56126 Pisa, Italy; daniela.monti@unipi.it (D.M.); valentina.paganini@phd.unipi.it (V.P.); susi.burgalassi@unipi.it (S.B.); patrizia.chetoni@unipi.it (P.C.); 2 Inter-University Center for the Promotion of the 3Rs Principles in Teaching and Research (Centro 3R), 56122 Pisa, Italy; 3 Global Medical Affairs Department, Almirall S.A., 08022 Barcelona, Spain; jordi.galvan@almirall.com; 4 Department of Translational Research and New Technologies in Medicine and Surgery, University of Pisa, 56126 Pisa, Italy; francesco.celandroni@unipi.it (F.C.); emilia.ghelardi@unipi.it (E.G.)

**Keywords:** ciclopirox, onychomycosis, bovine hoof membranes, nail lacquer, breathable cosmetic polish, transungual permeation, antimycotic activity

## Abstract

Background: Onychomycosis produces nail chromatic alterations that lead patients to mask them with cosmetic enamels. Objectives: Evaluate drug transungual permeation and antimycotic activity against selected strains after application of CPX-HPCH nail lacquer (NL) on the nail pre-covered with breathable cosmetic polish. Methods: CPX transungual permeation after applying CPX-HPCH NL once or twice a day on bovine hoof membranes pre-covered with a breathable cosmetic nail polish was compared to that obtained applying CPX-HPCH NL directly on the membrane. The relevant experimental permeates underwent an in vitro susceptibility test. Results: After CPX-HPCH NL application once a day, the drug transungual flux in the presence of cosmetic product tended to decrease while maintaining the antifungal activity. Two daily applications of CPX-HPCH NL on the membrane pre-covered with cosmetic polish exhibited the same permeation profile as daily application of the medicated lacquer directly on the nail as well as the same microbiological activity. Conclusions: The breathable cosmetic nail polish can be applied on the nail affected by onychomycosis in association with CPX-HPCH NL to mask the imperfections. The application of CPX-HPCH NL twice a day appears to be a good solution to obtain the same results as for a daily application without the presence of the cosmetic layer.

## 1. Introduction

Onychomycosis, one of the most common infections of the nail worldwide, is mainly caused by dermatophytes, molds, and yeasts [1] and can affect both fingernails and toenails. It manifests itself in nail changes such as unpleasant color, opacity, thickening or abnormal growth pattern, and damage to the nail plate [2,3] that are correlated to physical and psychological discomfort due to both the short- and long-term damage. Physical discomfort is often correlated with the progressing of the severity of the disease, while from a psychological point of view, this pathology can have an impact on the quality of life, including social, emotional, and functional aspects, related to the unpleasant appearance and pain, even if it is not life-threatening [4,5].

Depending on the severity of the nail condition and the type of fungus, different treatment protocols will be chosen that include, on one hand, mechanical and chemical treatments [6] and on the other hand, the use of antifungal drugs administered either topically, orally or a combination of the two. Among the antifungal drugs orally used, we can mention terbinafine and itraconazole that, despite the numerous side effects, represent the first choice in therapy. Amorolfine, ciclopirox, efinaconazole, and tavaborole are the drugs dedicated to topical treatment [7]. In this context, even the pharmaceutical form makes the difference, and in recent years formulations have been developed suitable for application on the nail, such as lacquers that can ensure better penetration of the drug across the nail plate by maintaining an appropriate drug concentration on the nail surface. Nail lacquers based on water-insoluble film-forming agents were the first medicated nail lacquers; they act as occlusive medications, such as a reservoir, releasing the drug that penetrates the nail over time, and are applied once a day or once a week, whereupon they must be removed with a solvent or nail file before the next application. Extensively used commercial products belonging to this category are based either on 8% ciclopirox, such as a US FDA approved formulation in 1999 for the management of mild-to-moderate dermatophytic onychomycosis or on 5% amorolfine, as a nail lacquer approved in Europe in 1991 for onychomycosis [7].

To counteract damage to the nail structure due to the removal of the formulation with solvents or nail files, new formulations containing water-soluble film-forming agents have been developed. Ciclopirox 8% HPCH nail lacquer is the main representative of this category, whose efficacy has been demonstrated in both preclinical [8,9,10] and clinical studies [11,12,13,14,15]. In recent years, other active principles against onychomycosis have been introduced on the market such as efinaconazole with potent antifungal activity (10% efinaconazole) and tavaborole, a boron-based small molecule, both marketed in 2014 [16,17]; the first is based on a mixture of alcohol and propylene glycol containing cyclomethicone, and the second one is based on a hydroalcoholic solution.

Onychomycosis is commonly long lasting and the therapy must automatically cover a period of several months, also considering that the complete regrowth of healthy toenails can take up to 18 months [18,19]. During this time, patients may choose to mask the chromatic alterations and/or the hyperkeratosis of the infected nails, with colored cosmetic enamel to improve the nail appearance during the long period of treatment. This would help patients to adhere to the therapeutic protocol without becoming discouraged if they do not appreciate progress relatively soon after the start of treatment. Thus, it became imperative to test the influence of cosmetic enamel applied simultaneously with medicated nail lacquer on the effectiveness of the pharmacological treatment.

This work was aimed at verifying the activity of ciclopirox after the application of ciclopirox HPCH nail lacquer (CPX-HPCH NL) when the nail is pre-covered with a cosmetic polish, to mask the condition of the nail from an aesthetic point of view and, at the same time, continue the antifungal therapy until the end of the treatment period to pursue the resolution of the disease. The transungual permeation of ciclopirox (CPX) after applying CPX-HPCH NL on bovine hoof membranes pre-covered with a commercial breathable cosmetic nail polish was evaluated and compared to that obtained applying CPX-HPCH NL directly on the biological membrane. In this research, a breathable cosmetic nail polish was chosen for its very rich composition acting as a base coat, color and topcoat, all in one, therefore in a condition in which the nail undergoes an integral cosmetic treatment and not a simple application of decorative/protective nail polish. In addition, breathable nail polishes are formulated, differently from traditional nail enamels, with a technology that allows water in and also lets moisture escape, leaving the nail clean and dry underneath [20].

## 2. Materials and Methods

### 2.1. Chemicals

The following products were used: ciclopirox (CPX, Erregierre SpA, San Paolo d’Argon, Italy); ciclopirox hydroxypropyl chitosan nail lacquer (CPX-HPCH NL, batch n. 21746a, 8% CPX, Almirall, Barcelona, Spain) and commercial breathable cosmetic nail polish (BreathCOSM, ORLY BREATH, NAMASTE HEALTHY, treatment + color, red color). All other chemicals and solvents were of analytical grade.

### 2.2. In Vitro Permeation Experiment

#### 2.2.1. Tissue Model

Bovine hoof membranes, although more permeable than human nails and less selective toward large permeants, are a commonly accepted and widely validated model for human nails and in vitro testing of medicated nail lacquers [21,22]. They have a less dense keratine network due to a significantly lower content of half-cysteine and disulphide linkages with respect to human nails; in any case, both substrates mainly consist of keratin, then the affinity of a penetrant for human nails and bovine hooves should be similar. Moreover, a parallelism between permeation through bovine hooves slices and human infected big toenails was demonstrated by Monti et al. [22], suggesting a substantial equivalence between both tissues.

Hooves from freshly slaughtered cattle, free of adhering connective and cartilaginous tissue, were soaked in water for 24 h; slices were cut from the distal part of the ball horn with a cryotome (2800 Frigocut E, Reichert-Jung).

Prior to the experiments, the bovine hoof membranes were soaked in water for 12 h to reduce rigidity and to give them a planer shape. The effective thickness of the hoof bovine membranes was determined after hydration. The membranes used in this study had a thickness of 148.4 ± 9.98 µm (±SE).

#### 2.2.2. Application of Cosmetic Nail Polish on Bovine Hoof Membranes

**Figure 1:**
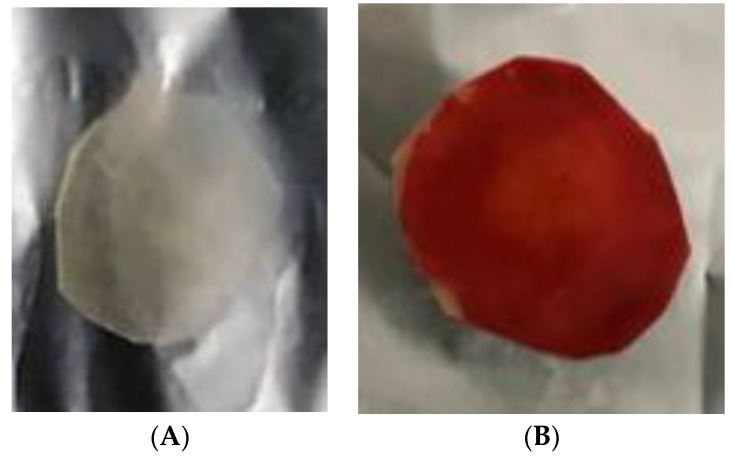
Bovine hoof membranes before (**A**) and after (**B**) treatment with commercial breathable cosmetic nail polish (red color).

The procedure for applying cosmetic nail polish to the bovine nail plates included different steps. The membranes were hydrated with distilled water overnight, to soften them and to facilitate the homogeneous distribution of the cosmetic lacquer on the nail. They were then wiped with absorbent paper and the cosmetic polish layer was applied and left in the air until completely dry. Then, the membranes were again hydrated overnight and subsequently inserted into the vertical diffusion apparatus after having measured the thickness again. The change in thickness was about 40 µm. Bovine nail plates without applying the cosmetic nail polish layer were used as reference. In Figure 1, bovine hoof membranes before (A) and after (B) treatment with commercial cosmetic nail polish are shown.

#### 2.2.3. Experimental Procedure

The permeation studies were performed using Gummer-type vertical diffusion cells and lasted 5 days.

The cell consisted of a donor and a receiving compartment with a volume of 1.0 and 5.0 mL, respectively. The membrane, pre-covered or not and with commercial breathable cosmetic nail polish, was interposed between the two compartments, and the area available for the permeation was 1.23 ± 0.99 cm^2^. A total of 10 μL of CPX-HPCH nail lacquer, withdrawn with a micropipette, and enough to cover the area available for permeation, was introduced into the donor compartment, and dried under a slightly heated air current to ensure the evaporation of the solvent and the formation of a film on the substrate. The amount of 10 μL was chosen because it corresponds to one application of the medicated nail lacquer with a brush during the treatment (data experimentally verified).

The application of the medicated lacquer was repeated daily (at 9:30 am) for 4 consecutive days after removing the residue of the previous application from the nail plate with a cotton swab soaked in water (Type A test). Moreover, the transungual permeation of ciclopirox after the application of CPX-HPCH NL directly in contact with the bovine hoof membrane was monitored.

In a second series of experiments, the medicated lacquer was applied twice a day at 9:30 am and 5:30 pm (Type B test).

The above-mentioned tests were also performed by applying only vehicle without CPX on the membrane pre-covered with the commercial breathable cosmetic nail polish to investigate both the presence of any interference on the drug permeation through the bovine hoof membrane and the drug analytical detection of the components of the cosmetic lacquer.

As the receiving phase, an isotonic phosphate buffer solution (PBS, 66.7 mM, pH 7.4) containing 0.003% w/v sodium azide to prevent bacterial growth was used. The receiving phase was stirred at a constant speed of 600 rpm and the temperature was kept constant at 37 ° C by circulating thermostated water in the double wall of the cell. At predetermined time intervals, samples of the receiving phase were withdrawn, replacing them with an equal volume of the fresh receiving phase to maintain the sink conditions.

Two different experimental protocols were adopted in terms of withdrawal times of the receiving phase: Type A test: 6, 20, 24, 30, 44, 48, 54, 68, 72, 78, 92, 96 h; Type B test: every 8 h for the first day and then every 4 h until the end of the experiment. When the HPCH nail lacquer was applied directly on the membrane, the withdrawal of the receiving phase took place every 4 h even in the first 24 h.

The amount of drug permeated was determined by HPLC analysis after the sample has been filtered (cellulose acetate filter, PHENEX RC 0.2 µm). All experiments were repeated six times.

### 2.3. Analytical Methods

The quantitative determination of CPX in the subungual fluid samples was carried out by HPLC. The apparatus consisted of a Shimadzu (Shimadzu Corp., Kyoto, Japan) LC-20AD system with an UV SPD-10A detector equipped with an autosampler SIL-10AD VP and a computer integrating system. The injection valve was a Rheodyne with a capacity of 20 µL. A Phenomenex Synergi^TM^ 4 µm Fusion-RP80Å, LC Column 150 mm × 4.6 mm was employed. The column was maintained at controlled temperature of 60 °C with a HPLC column temperature controller (Thermasphere^TM^, Phenomenex, Torrance, CA, USA). The mobile phase consisted of a mixture of acetonitrile: 20 mM phosphoric acid:methanol (50:30:20); the flux and the detection wavelength were 1.0 mL/min and 302 nm, respectively. The retention time was 4 min. The concentration of CPX in each sample was determined from a standard curve, obtained by plotting the concentration of known solutions (methanol solution of CPX diluted with receiving phase) vs. the corresponding peak areas of HPLC chromatograms. The influence of the cosmetic nail polish on the CPX quantitative analysis was also verified. In all cases, the cosmetic lacquer appeared not to interfere with the determination of the drug.

### 2.4. Data Analysis

A linear regression analysis of pseudo-steady state diffusion plots allowed for the calculation of the following parameters: steady-state flux (J), given by Q/A·t, where Q is the amount of permeant diffusing across the area A in time t; lag time, indicating the time taken by the drug to saturate the membrane and to reach the receiving phase, calculated from the X-axis intercept values of regression line; drug permeated at the end of experiment (Q_96h_).

All data are the average of six determinations ± standard error (SE). Statistical differences between permeation parameters were assessed by GraphPad Prism software (GraphPad Software Inc., San Diego, CA, USA). The evaluation included the calculation of means and standard errors, and group comparisons using the Student’s two-tailed unpaired *t*-test. Differences were considered statistically significant at *p* < 0.05.

### 2.5. Activity of Subungual Fluids against C. parapsilosis and T. rubrum

#### 2.5.1. Experimental Procedure

The in vitro antifungal activity of transungual permeates was qualitatively assessed against *Candida parapsilosis* (ATCC 22019) and *Trichophyton rubrum* (MYA 4438) by the broth microdilution methodology. Broth microdilution assays were performed in accordance with the guidelines of the Clinical and Laboratory Standards Institute (CLSI) M27-A4 for yeast and standard M38-A2 for filamentous fungi [23,24].

The medium used for broth microdilution susceptibility testing was RPMI 1640 medium (Gibco, Gran Island, NY, USA) containing L-glutamine and buffered at pH 7.0 with morpholinepropanesulfonic acid (MOPS) [25]. Serial 2-fold dilutions of the subungual fluids derived from 6 different permeation experiments for both Type A tests (withdrawn at 6, 20, 24, 30, 44, 48, 54, 68, 72, 78, 92, and 96 h) and Type B test (8, 16, 24, 28, 44, 48, 56, 68, 72, 80, 92, and 96 h) were prepared in RPMI 1640 medium according to the CLSI guidelines. Yeasts were grown on Sabouraud dextrose agar (SDA; bioMeriéux, Marcy l’Etoile, France) for 48 h at 30 °C and collected with sterile saline solution. The homogeneous yeast suspension was adjusted to 0.5 McFarland using a Densimat photometer (bioMeriéux, Marcy l’Etoile, France) and subsequently diluted in RPMI 1640 medium to a concentration of 0.5 × 10^3^ to 2.5 × 10^3^ colony forming units of (CFU)/mL. The stock inoculum suspensions of *T. rubrum* were prepared from 2-week-old fungal cultures in SDA. This strain well sporulated after this period. Suspensions were obtained by covering the fungal colonies with sterile saline solution and gently rubbing the colonies with the tip of a transfer pipette. The resulting conidial suspensions were transferred to sterile tubes. Collected conidia were allowed to settle for 10 to 15 min, counted using a hemocytometer, and then adjusted to 1 × 10^3^ to 3 × 10^3^ conidia/mL in RPMI 1640 medium. Plate counts were performed to verify the conidium concentrations by plating aliquots of the adjusted conidial suspensions on SDA. The fungal inocula were added to the microdilution plates. The antimicrobial activity of transungual fluids was assessed if producing at least 80% growth inhibition.

#### 2.5.2. Efficacy Index

To predict the topical efficacy of ciclopirox permeated through the bovine hoof membranes pre-covered with the cosmetic lacquer, it is important to relate the amount of drug permeated over time and the sensitivity of the fungi to the antifungal drug under study. An efficacy index (EI) [8,21], defined as the ratio between the expected drug concentration at the site of action after 1 day of application, and the minimal inhibitory concentration (MIC) against each tested strain, was determined. The higher the EI, the better in vivo treatment efficacy could be expected.

EI was calculated as a ratio of flux of antifungal agent through hoof slices to the minimal inhibitory concentration (µg/cm^2^ day/MIC) of the drug against the tested strains, considering a hypothetical permeation unit (1 cm^2^ per 1000 μm) and a subungual volume equal to 1.0 mL.

The MIC values of CPX were 0.312 μg/mL and 0.625 μg/mL against *T. rubrum* and *C. parapsilosis,* respectively.

## 3. Results

### In Vitro Permeation Experiments

**Figure 2:**
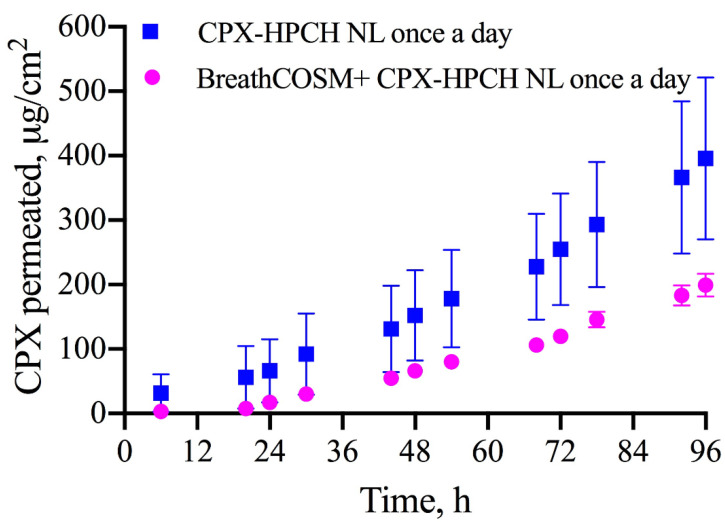
Permeation plot of CPX through bovine hoof membranes after application once a day of CPX-HPCH nail lacquer on the nail, pre-covered or not with commercial breathable cosmetic nail polish, by using Type A protocol.

The relevant permeation parameters (flux, lag time, and CPX amount at the end of the experiments) after the application of HPCH nail lacquer once a day (Type A test) on the bovine hoof membranes pre-covered or not and with commercial breathable cosmetic nail polish, are summarized in Table 1, while the relative permeation plots are graphically shown in Figure 2. It is possible to notice a trend of a decrease in the amount of permeated drug over time when the cosmetic product is interposed between the medicated nail lacquer and the bovine hoof membrane, although the differences in the permeation parameters are not statistically significant.

The subungual permeates, collected from Gummer-type permeation cells at 6, 20, 24, 30, 44, 48, 54, 68, 72, 78, 92, and 96 h after application of 10 µL of CPX-HPCH nail lacquer on the bovine hoof membranes, pre-covered or not with cosmetic nail polish, underwent a antifungal susceptibility test. The minimal amount of fluid required to inhibit fungal growth for each strain tested is reported in Table 2: the antifungal activity against *C. parapsilosis* and *T. rubrum* was demonstrated for all the samples collected during the entire period of the experiment (6–96 h). EI are definitely above 1 with greater activity if the medicated lacquer is applied directly to the nail, without statistical difference (EI = 19.89 ± 5.25 and 39.84 ± 10.71 against C. parapsilosis and T. rubrum strains, respectively. In the case of no cosmetic polish, EI = 9.78 ± 0.85 and 19.58 ± 1.70 towards *C. parapsilosis* and *T. rubrum*, respectively; in presence of breathable cosmetic product, Table 3).

The second part of the work had the aim of verifying whether the application of CPX- HPCH nail lacquer twice a day (Type B test) on the bovine hoof membrane pre-covered with commercial breathable cosmetic nail polish was allowed to have a transungual permeation comparable to that obtained by applying the medicated lacquer directly on the nail once a day. These additional experiments were undertaken on the basis of the results of the above-described first phase of this work, which demonstrated the remarkable role of the cosmetic lacquer to act as a barrier layer hindering the transungual permeation of CPX, after the application of CPX-HPCH NL. Indeed, it should be emphasized that the application of the cosmetic lacquer involves a change in the barrier thickness of about 40 microns.

This second set of experiments was performed by adopting an experimental protocol, which provided for more frequent sampling of the receiving phase (every 8 h for the first 24 h and every 4 h until the end of the experiment; Type B test). The experimental protocol was modified to prevent the multiple application of medicated nail lacquer from hindering the diffusion process through the barrier formed by the biological membrane and cosmetic enamel. The suitable experimental condition is chosen based on the amount of drug applied, which allows for maintaining the sink conditions and the maximum difference in concentration between the donor and recipient compartment. Permeation occurs by diffusion, which is regulated by the difference in concentration between the two sides of the barrier.

**Figure 3:**
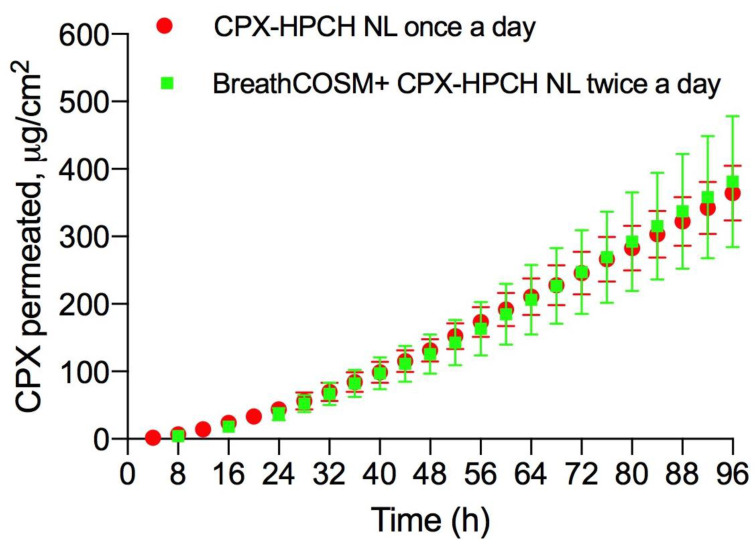
Permeation plot of CPX through bovine hoof membranes protocol after application twice a day of CPX-HPCH NL on the nail pre-covered with commercial breathable cosmetic nail polish compared to the application of CPX-HPCH NL once a day directly on the nail by using Type B protocol.

The relevant permeation parameters (flux, lag time, and CPX amount at the end of the experiments) are summarized in Table 4, while the permeation plot of CPX is graphically illustrated in Figure 3.

Two daily applications of 10 µL of CPX-HPCH nail lacquer on the bovine hoof membrane pre-covered with cosmetic polish exhibited the same permeation profile as 10 µL daily application of medicated lacquer directly on the nail (Figure 3). The permeation parameters also confirm what is graphically evident (Table 4).

Only the lag time appears to increase reaching 20.19 h, presumably due to the presence of the cosmetic coating, which acts as an additional barrier, even if the differences are not statistically significant.

Moreover, in this second set of experiments, the subungual permeates were subjected to an antifungal susceptibility test. The antifungal activity against the strains tested (*C. parapsilosis* and *T. rubrum*) was demonstrated for all the samples collected.

EI values towards both *C. parapsilosis* and *T. rubrum* when the HPCH nail lacquer was applied on the bovine hoof membranes, pre-covered with cosmetic product twice a day, were similar (p = 0.6757) to those obtained after the application of medicated nail lacquer once a day directly on the nail (Table 5).

In addition, it is noteworthy that the application of medicated nail polish both once a day and twice a day does not alter the appearance of the cosmetic nail polish and no color transfer to the applicator brush was highlighted.

## 4. Discussion

This topic has been addressed to answer the growing demand to camouflage the condition of a nail affected by onychomycosis as much as possible, in order to have a social life without conditioning. In recent years, this topic gained scientific resonance and many researchers studied the influence of cosmetic treatment on the effectiveness of pharmacological treatment of the infected nail. Lately, scientific research has focused on demonstrating the possible coexistence of cosmetic and pharmacological nail treatments without reducing the effectiveness of the therapies, looking for the right administration protocol for this purpose. In particular, the existing literature in this context concerns amorolfine 5% nail lacquer (NL) and topical solutions of tavaborole and efinaconazole. Table 6 summarizes the experimental conditions of the studies performed by the different research groups on these drugs. Sigurgeirsson et al. [26] have demonstrated that the application of the cosmetic nail varnish does not affect the antifungal efficacy of amorolfine 5% nail lacquer in the treatment of distal subungual toenail onychomycosis. This clinical study compared the therapeutic effect on two groups of patients, one treated for 12 weeks with amorolfine 5% nail lacquer alone, the other with amorolfine 5% nail lacquer plus a cosmetic nail varnish; the latter applied 24 h after the medicated lacquer. Both groups received once a week topical administration of amorolfine 5% NL; 24 h later, the group selected for the cosmetic treatment received the cosmetic nail vanish, applying it evenly over the medicated nail polish. At the end of the treatment period (12 weeks), the antifungal activity of the distal part of clipped infected nails was evaluated by measuring the zones of inhibition on agar plates seeded with *T. mentagrophytes*. All subjects in both groups had negative mycological cultures for dermatophytes and non-dermatophyte pathogens. The study continued with the in vitro evaluation, using 11 different brands of cosmetic nail polishes of different origin and composition on cadaver nails and adopting the same application protocol of medicated and cosmetic products and the microbiological test on *T. rubrum* [27]. The results obtained confirmed that the cosmetic lacquers applied over the medicated lacquer do not affect the transungual permeation of amorolfine. It can be assumed that there is no interaction between medicated and cosmetic lacquer, at least by adopting this application protocol. In the last revision of the leaflet of commercial product containing 5% amorolfine, which dates back on July 2017, the following recommendation was introduced: “cosmetic nail varnish can be used, but you should wait at least 10 min after applying medicated product before painting your nails”, probably based on the results obtained by these studies.

As regards efinaconazole, there are three different studies that have dealt with the influence of cosmetic lacquers on the efficacy and compatibility of a 10% topical solution. In 2014, Zeichner et al. [28] evaluated the penetration of radiolabelled efinaconazole through human nails coated with cosmetic products of different brands using an experimental formulation of (^14^C)-efinaconazole that was prepared to reach a concentration of 0.15 microCi/dose. The administration of the medicated product was daily and the experiment lasted 7 days. Both permeation and penetration did not appear to be affected by the cosmetic enamel coating, although, as the authors themselves report, the study presents several obscure points, such as the fact that the non-use of cosmetic lacquers in conjunction with the medicinal product is still reported in the package leaflet and that the microbiological efficacy has not been demonstrated. Furthermore, a change in the appearance of the cosmetic layer in terms of the loss of color and stickiness following the repeated application of the product containing efinaconazole was highlighted; the same manifestations were also observed by Vlahovic et al. [19] in a similar study. In a clinical study on 13 patients, lasting 52 weeks, Canavan et al. [29] evaluated the efficacy of 10% efinaconazole solution with the use of nail polish in the therapy of distal lateral subungual onychomycosis for 52 weeks by measuring, on the one hand, nail growth and thickness, and on the other, the compatibility by a questionnaire. Moreover, in this case, no differences between the two treatments in the presence and in the absence of cosmetic polish have been detected in the onychomycosis severity index, calculated by taking in the account of the change of nail involved and the degree of infection. The negative effect, which made the patients unsatisfied and limited compliance, was concerned in the appearance of the cosmetic polish that was losing color and presented a tacky texture, also suggesting a lack of integrity of the barrier represented by the layer of cosmetic enamel interposed between the antifungal product and the nail. These alterations can be compatible with the presence, in the formulation of the commercial product based on efinaconazole, of excipients such as the C_12_-C_15_ alkyl lactate, which is also widely used in the cosmetic field for its wetting and dispersing properties for pigments, of which cosmetic nail polish is very rich.

Vlahovic et al. [16] tested in vitro the behavior of the topical solution of 5% tavaborole applied daily on cadaver nails previously treated with one or more layers of commercial cosmetic polish. The experiments lasted 20 days. The researchers reported that the application of nail polish did not limit the nail penetration of tavaborole. It is known that tavaborole had better nail penetration than other antimycotic drugs, such as amorolfine and ciclopirox, due to their low molecular weight and hydrophilic nature [30]. These characteristics can be thought to positively influence transungual permeation even in the presence of a cosmetic nail treatment.

In a later work [19], the same authors have reported that the treatment with tavaborole formulation did not produce any change in the appearance of the polished nails; the cosmetic enamel layer remained intact, without the phenomena of discoloration or color transfer.

On the basis of the data collected in recent years about the combination of cosmetic treatment and pharmacological treatment for the cure of onychomycosis, in this work the behavior of the ciclopirox HPCH nail lacquer was examined in terms of transungual permeation and microbiological activity of the drug when the medicated nail lacquer was applied to the nail pre-covered with a cosmetic polish. Bovine hoof membrane was used as a substrate. The commercial breathable cosmetic nail polish was chosen, as it represents an integral cosmetic treatment for the nail, acting as a base coat, color (red), and topcoat, all in one. Furthermore, unlike the traditional nail enamels, formulated with tightly stacked molecules, with particles virtually fused together once the polish is dry, breathable nail polishes keep polymer molecules suspended in a brick-like pattern and, when the nail polish is rubbed, the suspended molecules move just enough to allow O_2_ and water particles through [20].

The presence of cosmetic nail polish led to a decrease in the amount of drug permeated over time when 10 microliters of ciclopirox HPCH nail lacquer was applied once a day on the bovine nail plate pre-covered with the cosmetic product. Despite this, the drug quantified in the receiving phase at all times of the experiment (from 6 h after administration up to 96 h, corresponding to the end of the permeation experiment) is sufficient to exert the antifungal activity against *C. parapsilosis* and *T. rubrum,* with an efficacy index (EI) definitely higher than 1, in particular 9.78 ± 0.85 and 19.58 ± 1.70, respectively, suggesting that the formulation is active even when a double barrier consisting of both nail plate and cosmetic nail polish has to be overcome.

The change from 1 to 2 times a day in the administration mode of ciclopirox HPCH nail lacquer formulation on the bovine nail plate pre-covered with commercial breathable cosmetic nail polish, produced a CPX transungual flux comparable to that obtained after the application of medicated nail polish once a day directly on the nail plate: 4.97 ± 1.28 vs. 4.37 ± 0.58 µg/cm^2^h, respectively. The application of CPX-HPCH nail lacquer twice a day appears to be a good solution to obtain the same results as those derived from a daily application without the presence of the cosmetic layer, considering there is a correlation between the amount of formulation applied and the amount of drug permeated, even if there is not a strictly direct proportionality. Furthermore, by correlating the drug concentration at the site of action, which is directly proportional to its transungual flux, and the susceptibility of the fungi to the antifungal agent, we obtain superimposable EI values, and expect a similar therapeutic effectiveness.

## Figures and Tables

**Table 1:** Permeation parameters of CPX through bovine hoof membranes (flux, lag time, and amount of drug permeated at the end of permeation experiment, 96 h, Q_96h_) by using Type A protocol (mean ES, n = 6).

Membrane Treatment	CPX-HPCH Nail Lacquer Applications Number (10 µL)	Flux (J, µg/cm^2^ h)	Lag Time (h)	Q_96h_ (µg/cm^2^)	Experimental Procedure
No cosmetic polish	Once a day	5.18 ± 1.39	22.88 ± 0.91	379.10 ± 101.8	Type A
BreathCOSM, 1 coat	Once a day	2.55 ± 0.22	20.28 ± 1.18	195.7 0 ± 15.69	Type A

**Table 2:** Minimal inhibitory volumes required to inhibit *Trichophyton Rubrum* and *Candida parapsilosis* growth when CPX-HPCH nail lacquer was applied once a day on the bovine hoof membrane coated with commercial breathable cosmetic nail polish by using experimental conditions of Test A.

Collection Time (h)	Minimal Inhibitory Volume, µL	
	*Candida parapsilosis*	*Trichophyton rubrum*
6	100	100
20	25	25
24	25	50
30	25	25
44	25	25
48	25	25
54	25	25
68	12.5	12.5
72	25	25
78	25	12.5
92	25	6.25
96	25	25

**Table 3:** Efficacy index of CPX after application once a day of CPX-HPCH nail lacquer on the nail, pre-covered or not with commercial breathable cosmetic nail polish, by using Type A protocol (mean ±SE, n = 6).

Membrane Treatment	CPX-HPCH Nail Lacquer Applications Number (10 µL)	*Candida parapsilosis*	*Trichophyton rubrum*	Experimental Procedure
No cosmetic polish	Once a day	19.89 ± 5.35	39.84 ± 10.71	Type A
BreathCOSM,1 coat	Once a day	9.78 ± 0.85	19.58 ± 1.70	Type A

**Table 4:** Permeation parameters of CPX through bovine hoof membranes (flux, lag time, and amount of drug permeated at the end of permeation experiment, 96 h, Q_96h_) by using Type B protocol (mean ± ES, n = 6).

Membrane Treatment	CPX-HPCH Nail Lacquer Applications Number (10 µL)	Flux, J, µg/cm^2^ h	Lag Time, h	Q_96h_ µg/cm^2^	Experimental Procedure
No cosmetic polish	Once a day	4.37 ± 0.58	14.66 ± 2.96	352.70 ± 42.35	Type B
BreathCOSM, 1 coat	Twice daily	4.97 ± 1.28	20.19 ± 1.03	375.0 ± 94.65	Type B

**Table 5:** Efficacy index of ciclopirox after application twice a day of CPX-HPCH NL on the nail pre-covered with commercial breathable cosmetic nail polish compared to the application of CPX-HPCH NL once a day directly on the nail by using Type B protocol (mean ± SE, n = 6).

Membrane Treatment	CPX-HPCH Nail Lacquer Applications Number (10 µL)	*Candida parapsilosis*	*Trichophyton rubrum*	Experimental Procedure
No cosmetic polish	Once a day	16.77 ± 2.21	33.58 ± 4.43	Type B
BreathCOSM, 1 coat	Twice daily	19.09 ± 4.90	38.22 ± 9.82	Type B

**Table 6:** Experimental conditions of the studies performed by the different research groups on amorolfine, efinaconazole, and tavaborole.

StudyType	Medicated NL Application		Treatment Length	Influence of Activity	Cosmetic Layer Appearance	Reference
	Before Cosmetic NP	After CosmeticNP				
Amorolfine 5%						
In vivo	24 h	-	12 weeks	No difference in antimycotic activity	-	[26]
In vitro Human nail	24 h	-	Immediately after drying	No difference in antimycotic activity	-	[26]
In vitro Human nail	10 min	-	Immediately after drying	No difference in antimycotic activity	-	[26]
Efinaconazole 10%						
In vitro Human nail		daily	7 days	No difference in permeation/penetration	Loss of color and stickiness after repeated application	[28]
In vitro Human nail		daily	7 days	-	Discoloration	[19]
In vivo		daily	52 weeks	No differences in onychomycosis severity index	Discoloration Tacky texture	[29]
Tavaborole 5%						
In vitro Human nail		daily	20 days	No difference in penetration		[16]
In vitro Human nail		daily	7 days	-	No discoloration	[19]

NL: nail lacquer; NP: nail polish.
